# Time-managed PAPR use enables a balanced approach to infection control and personal freedom

**DOI:** 10.1038/s41598-025-32625-3

**Published:** 2025-12-13

**Authors:** Yusaku Fujii

**Affiliations:** Gunma University, Kiryu, Japan

**Keywords:** PAPR for everyone, Powered air-purifying respirator (PAPR), Engineering alternative to lockdown, Saved allowance time (SAT), Airborne infectious disease, Non-pharmaceutical intervention (NPI), Verifiable record of AI output, PAPR wearing-status networked management system (PWS-NET), Engineering, Health care, Mathematics and computing

## Abstract

Full lockdowns during airborne-disease pandemics impose substantial socio-economic costs. To address this, to the best of the author’s knowledge, three prior contributions were made for the first time: (i) proposed a concept in which medical-grade Powered Air-Purifying Respirators (PAPRs) used by the general public can serve as an engineering alternative to lockdowns; (ii) disclosed a proof-of-concept PAPR with aerosol-blocking performance comparable to medical devices at a parts cost of USD 40; and (iii) demonstrated via mathematical modeling that if more than 55% of the population wears PAPRs continuously—or if everyone wears them intermittently to a moderate extent—the effective reproduction number ***R***_**t**_ can be reduced from 2.0 to 0.9. Building on these prior results, this study proposes and prototypes an IoT-based management framework—the PAPR Wearing-Status Networked Management System (PWS-NET)—that seeks to reconcile governmental infection control with individuals’ freedom to choose the time and place of non-wearing. The core metric is Saved Allowance Time (SAT), i.e., an accumulative daily allowance for mask-off periods. The prototype integrates three components: (a) real-time wearing detection for PAPRs using a differential-pressure sensor, (b) user-declared location via a smartphone application, and (c) a rule-based web server that updates SAT on a daily basis. Scenario tests that emulate realistic use conditions confirmed correct operation of SAT updates and violation judgments, as well as effective real-time visual feedback to users. Constructed entirely from off-the-shelf components, the prototype is intended as a starting point for large-scale field studies aimed at integrating SAT-based governance into public-health policy for future outbreaks.

## Introduction

In pandemics caused by airborne infectious agents^1^, non-pharmaceutical interventions (NPIs), such as lockdowns, social distancing, school closures, remote work, restrictions on domestic and international travel, mandatory mask-wearing, isolation, and contact tracing, have been deemed essential. At the same time, it is widely recognized that such NPIs impose substantial social and economic burdens^2–6^. Among them, lockdowns are effective in suppressing transmission but severely limit economic activity and personal freedom, and are not necessarily sustainable^7–10^. The author has proposed the use of powered air-purifying respirators (PAPRs) by the general public as an engineering alternative to lockdowns^11,12^.

This study is based on the premise that continuous mask-wearing is already a socially accepted behavior, and positions PAPRs as an extension of that practice. It also assumes, in line with the prediction that another pandemic is inevitable^13^, that among the various transmission routes, airborne transmission—similar to COVID-19—will again be a critical challenge not easily prevented by conventional NPIs^14,15^. The infectious dose for COVID-19 has been reported to be 300–2000 virions^16^, and assuming a similar dose for the next pandemic, it is considered feasible to reduce infection risk by decreasing the number of aerosolized virions inhaled through the use of PAPRs^17^.

PAPRs are respiratory protection devices that combine air purification through high-performance filters such as HEPA^18,19^ and low breathing resistance achieved by powered airflow. Originally developed for healthcare workers and industrial personnel operating in high-risk aerosol environments^20–22^, commercial medical-grade PAPRs (e.g., those manufactured by 3M^23,24^ and CleanSpace^25,26^ typically feature simple structures that achieve a high level of protection, with assigned protection factors reaching 1000^27^. Medical-grade PAPRs provide the following air purification functions:

1. HEPA filters, which capture more than 99.97% of aerosols at the most penetrating particle size (0.3 μm)^28^, effectively block virus-containing aerosols in ambient air.
2. The hood is maintained at positive pressure via the supply fan, which prevents unfiltered outside air from entering through gaps between the hood and the face.
3. As a result of (1) and (2), the only air entering the hood—excluding exhaled breath—is highly purified via the HEPA filter.

This mechanism, which ensures a high level of air purification, consists of only a few basic components: a HEPA filter, a powered fan, a battery, and a hood capable of maintaining moderate airtightness and positive pressure. By contrast, conventional facemasks create negative pressure during inhalation, allowing unfiltered air to enter through gaps—this is their primary limitation as respiratory protection devices.

The author has proposed a novel application of medical PAPRs for use by the general public as a social-level infection control measure that could replace lockdowns^11^. In particular, prior work demonstrated that a PAPR with filtration performance equivalent to medical-grade devices—composed of a HEPA filter, powered fan, battery, and a hood with moderate positive pressure—can be self-built using parts costing approximately USD 40. During the COVID-19 pandemic, methods for fabricating PAPRs using 3D printers and safety evaluations were also proposed by research teams in the United States^29^.

Moreover, mathematical modeling has shown that strict, continuous PAPR usage by all individuals is not necessary to achieve effective transmission control^12^. For example, even under conditions where the effective reproduction number ***R***_**t**_ is 2.0, ***R***_**t**_ can be reduced to below 0.9 if approximately 55% of the population wears PAPRs continuously. Alternatively, equivalent results can be achieved if the entire population wears PAPRs intermittently such that the average probability of airborne infection is reduced by 55%.

Assuming perfect shielding performance by PAPRs, continuous use could theoretically prevent airborne transmission entirely. Under such conditions, protecting just 50% of the population with PAPRs would reduce ***R***_**t**_ from 2.0 to 1.0. This implies that universal and constant PAPR use is excessive and that partial adoption could suffice for epidemic control.

These findings support the feasibility of “minimally obligatory, selectively flexible deployment” strategies as a tolerable form of infection control. As a technical foundation for such a policy model, we have developed a prototype system called the PAPR Wearing-Status Networked Management System (PWS-NET), which (i) detects PAPR wearing status in real time, and (ii) enables fair allocation and management of non-wearing allowances on an individual basis. The system introduces “Saved Allowance Time (SAT)” as a time-based metric for managing non-wearing allowances on a daily basis, integrating (a) sensor-based detection of wearing status, (b) self-declared location reporting via smartphone, and (c) a rule-based algorithm implemented on a web server for dynamic SAT management.

This study aims to validate the basic functionality and feasibility of the system by building a working prototype and conducting scenario-based bench tests. Specifically, this paper discusses: (i) the design concept of PWS-NET as a socio-technical system based on SAT, (ii) the system architecture of the prototype, (iii) performance verification under realistic behavior scenarios, and (iv) potential applications of PWS-NET as a preparedness strategy for future airborne pandemics.

## Concept of PAPR wearing-status networked management system (PWS-NET)

This study proposes the concept of the PAPR Wearing-Status Networked Management System (PWS-NET), designed to achieve both respect for the individual’s right to choose when and where not to wear a PAPR, and the efficiency of government-led infection control.

The PWS-NET comprises the following functions:

1. Allowance Time (AT), representing the permitted non-wearing duration, is given daily (e.g., at 3:00 a.m.) by the government to each citizen. Each individual may accumulate AT up to a defined upper limit (e.g., AT × 7) as Saved Allowance Time (SAT), and may freely exercise their right to go unmasked in non-Certified Safe Zones (e.g., restaurants or party venues) within the limits of their SAT.
2. The wearing status (St-1) is automatically determined by a controller and sensor installed in the PAPR, and is transmitted to the user’s smartphone app. The system distinguishes St-1 = 0 (Non-wearing) and St-1 = 1 (Wearing).
3. The user declares their location status (St-2) via the smartphone app interface. The app sends both the automatically detected Wearing Status (St-1) and the self-declared Location Status (St-2) to a web server, and displays the current SAT value returned from the server.
4. The web server receives AT periodically from the government, and based on the received values of St-1 and St-2, dynamically updates and manages SAT, transmitting the updated value back to the smartphone app.

Three categories of Location Status (St-2) are defined: (a) Certified Safe Zone (CSZ, such as home), (b) Outside CSZ (e.g., public indoor or outdoor spaces), and (c) SAT Utilization/Exemption Mode. Within CSZs, mask-wearing is not required. Outside CSZs, PAPR wearing is mandatory, but the user may opt to go unmasked by consuming SAT. While St-2 is self-reported, the system presumes that healthy operation can be maintained by introducing spot checks for non-compliance and imposing strict penalties for false declarations.

**Fig. 1:**
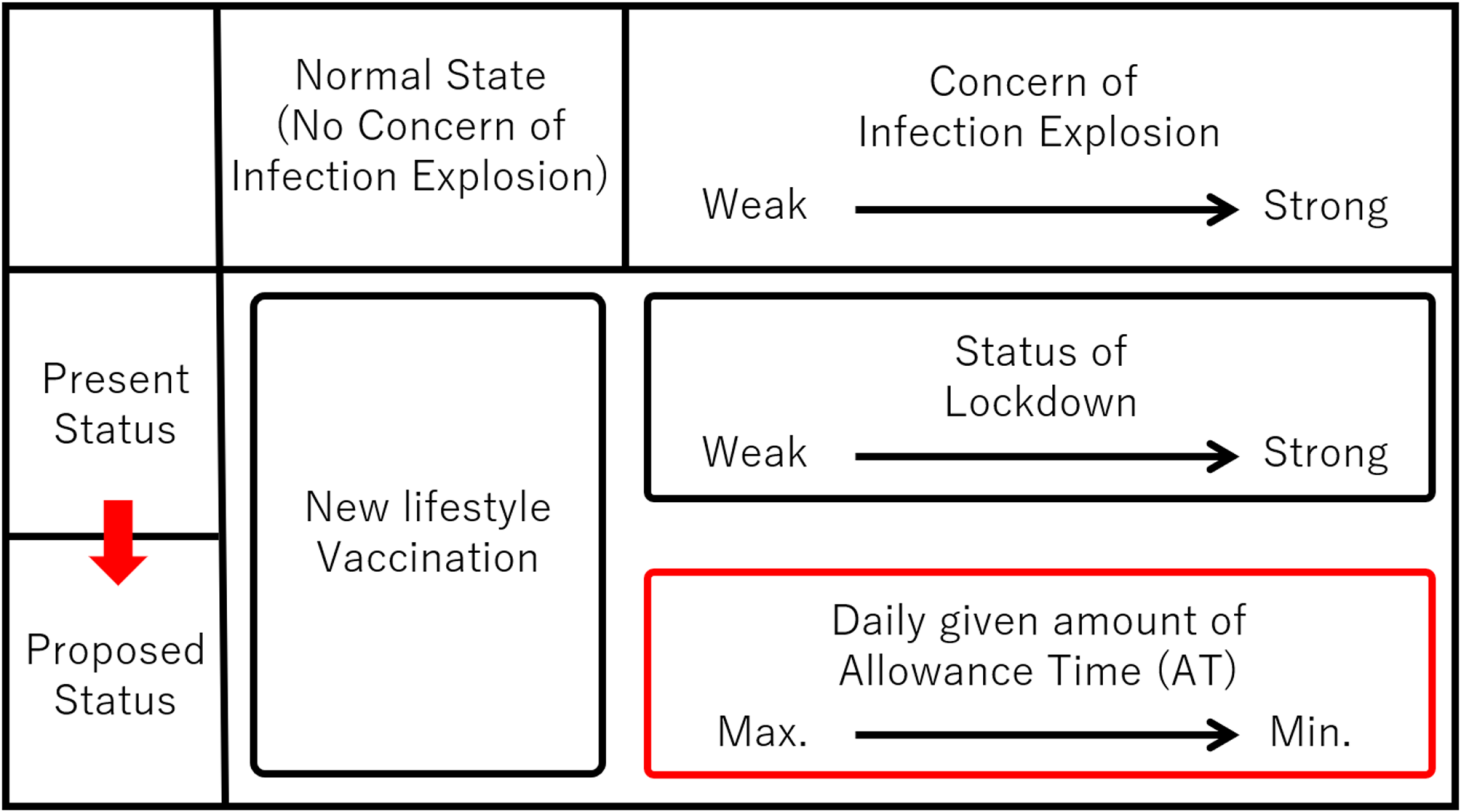
Policy framework of allowance time (AT) and the transition from the current model.

Figure 1 presents a conceptual diagram showing how the current infection control framework could be transformed into the proposed system in response to the degree of concern about infection spread. The horizontal axis represents the level of concern regarding infection explosion, and the structure is such that the intensity of lockdown measures changes accordingly. “New Lifestyle” and “Vaccination” appear at the center of the diagram as shared elements between the current and proposed systems.

In the current system, as concern increases, the government has no option but to tighten lockdown measures, imposing strict restrictions on personal movement. In contrast, under the proposed system, even during lockdown, individuals are allowed to go outside if wearing a PAPR, and furthermore, may remove the PAPR at self-selected times and locations within the limit of their SAT, which is accumulated from daily-allocated AT.

The red arrow in the figure indicates the shift from the current restrictive model to a flexible SAT-based self-governance model. Since AT can be adjusted depending on infection trends, this system aims to realize a new governance framework that balances infection control and personal liberty. The government can determine AT allocations based on present and future infection forecasts. If an unexpected surge in infections occurs, AT can be immediately reduced. At such times, the maximum SAT (e.g., AT × 7) would also be adjusted downward. For example, setting AT to zero would mean all citizens must wear PAPRs at all times when outside the home, enabling rapid suppression of transmission. By having this enforcement mechanism always in reserve, the government can experiment with more flexible policies with confidence.

This figure illustrates the transition in infection control frameworks according to the degree of concern about infection spread, from the current lockdown-centered system to the proposed SAT-based flexible governance model. Under the current system, as the infection situation worsens, the intensity of lockdown increases, and individual activities are uniformly restricted. In contrast, under the proposed system, individuals are allowed to go outside while wearing a PAPR, and furthermore, may select times and locations to temporarily go unmasked within the range of Saved Allowance Time (SAT), which is accumulated from daily allocated Allowance Time (AT). The red arrow indicates this transition toward a more flexible mode of self-governed operation.

## Prototype of PAPR wearing-status networked management system (PWS-NET)

**Fig. 2:**
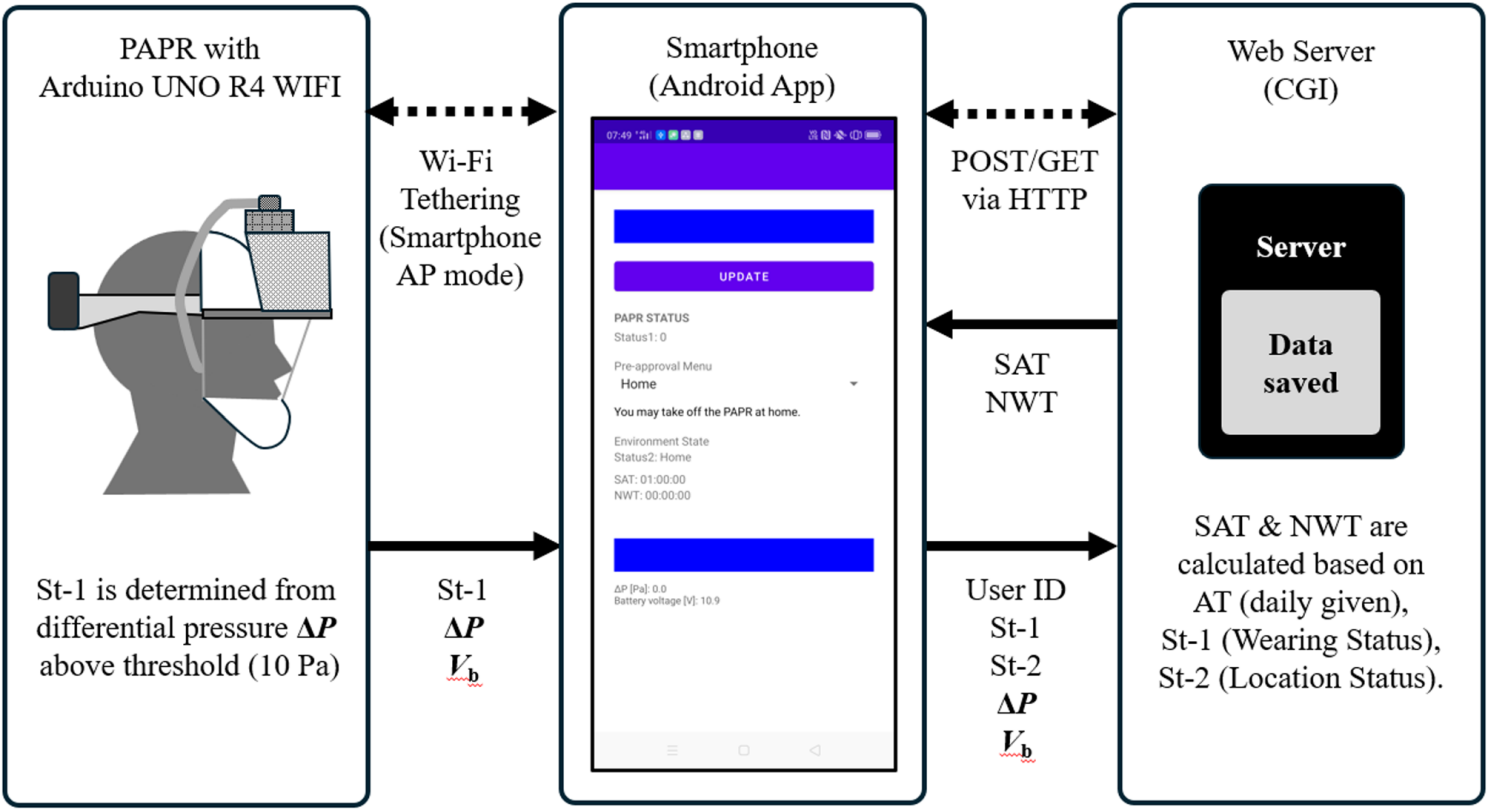
Overview of the three components comprising PWS-NET and their respective functions.

**Fig. 3:**
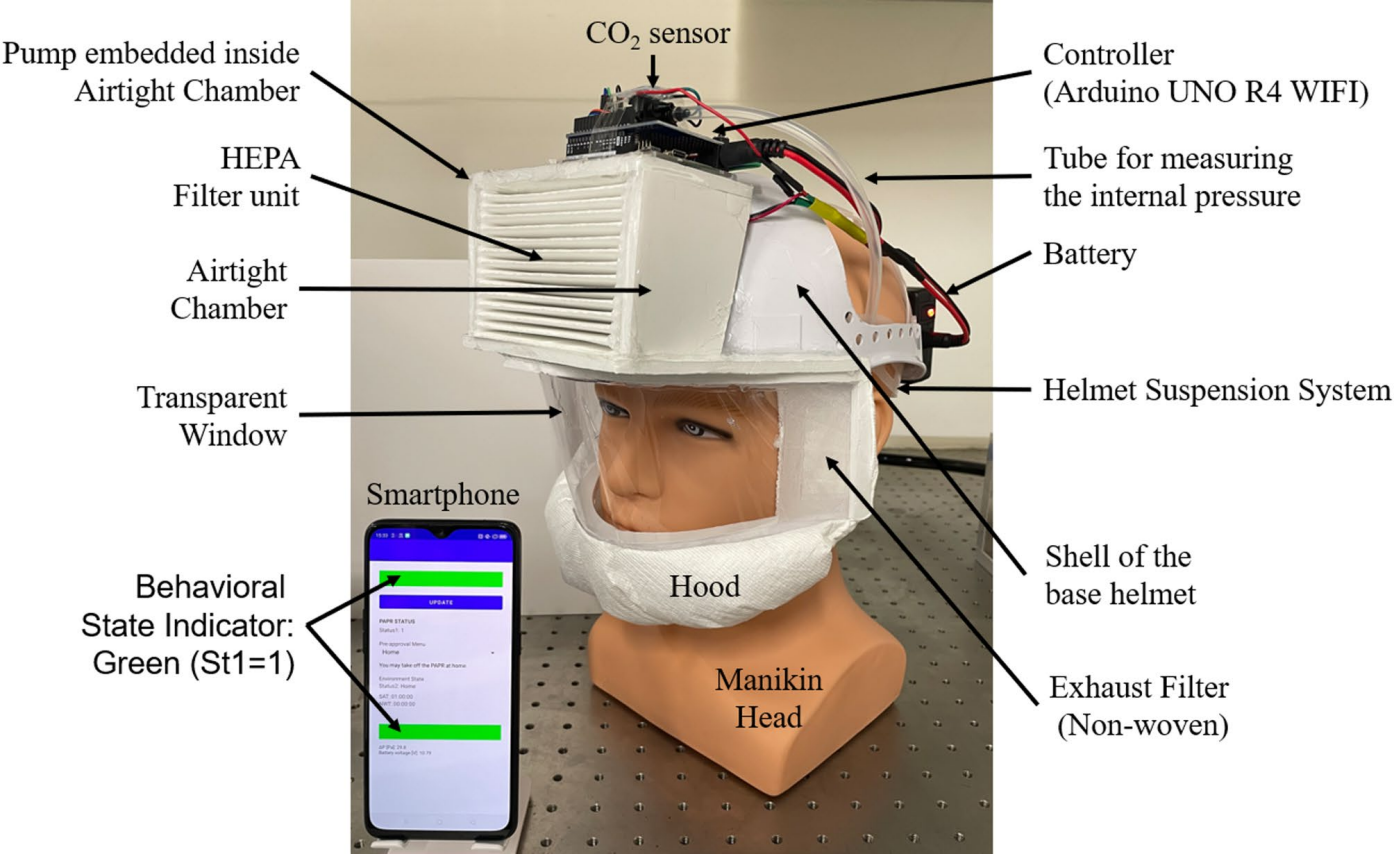
Appearance of the prototype PAPR and the smartphone application.

This study developed a prototype of the PAPR Wearing-Status Networked Management System (PWS-NET), with the aim of balancing infection control and personal freedom during airborne infectious disease pandemics. Figure 2 provides an overview of the three components of PWS-NET—PAPR, smartphone, and web server—and their respective functions. Figure 3 shows a prototype PAPR mounted on a mannequin head, along with the display screen of the smartphone application.

This figure illustrates the three components that constitute the proposed PWS-NET system: [a] a PAPR, which detects the wearing status in real time; [b] a smartphone, which transmits wearing and location information and provides visual feedback to the user; and [c] a web server, which calculates and manages Saved Allowance Time (SAT) and Non-wearing Time (NWT) based on a rule-based protocol.

### Powered air-purifying respirator (PAPR)

The prototype PAPR employs a light-duty helmet (shell plus suspension system) onto which a HEPA filter, fan, battery, and controller are mounted, forming a helmet-integrated PAPR unit (Fig. 3). The fan (model: San Ace B76, manufacturer: Sanyo Denki Corp., maximum static pressure: 150 Pa; maximum airflow: 360 L/min) is installed inside an airtight chamber made of styrofoam board. Clean air is drawn into the chamber via a HEPA filter installed at the front of the chamber, due to the negative pressure generated by the fan. The HEPA filter is cut from a commercially available air purifier filter (model: PMMS-DCHF, manufacturer: Iris Oyama Ltd.). The purified air introduced into the chamber is forcibly delivered into the hood by the fan. The fan is directly powered by a lithium-ion battery (model: DC12400, no-brand, sold via Amazon Corp., output voltage: 12.6–10.8 VDC; capacity: 4000 mAh; mass: 176.4 g).

This forced air inflow keeps the interior of the hood at positive pressure. As a result, airflow through any gaps between the hood seal and the user’s face is always directed outward, effectively preventing direct intrusion of unfiltered ambient air into the hood. The total weight of the prototype, including the battery, was 631.0 g, and the continuous operating time was approximately 330 min (5 h and 30 min). Since the operating time depends on the battery capacity, it can be extended in the future by adopting higher-capacity batteries.

The controller (model: Arduino UNO R4 WIFI, manufacturer: Arduino Corp.) measures the pressure difference between the inside and outside of the hood using a differential pressure sensor (model: SDP816-500 Pa, manufacturer: Sensirion). It also measures the battery voltage by using four 10 kΩ resistors connected in series to form a 1/4 voltage divider, with the resulting analog signal input to the controller. The wearing status (St-1) is determined as follows: if the differential pressure is less than 10 Pa, the system registers non-wearing (St-1 = 0); if it exceeds 10 Pa, wearing is assumed (St-1 = 1). The measurement loop operates at 10 ms intervals.

The controller connects to the smartphone’s Wi-Fi access point via tethering and sends a dataset—comprising differential pressure (**Δ*P***), battery voltage (**V**_**b**_), and wearing status (St-1)—in JSON format every 2 s.

### Smartphone

The smartphone (model: CPH1983, manufacturer: Oppo Corp., OS: Android ver. 9) runs a custom JavaScript-based application. The app receives the dataset (St-1, **Δ*P***,) from the PAPR controller and appends the user’s ID (pre-registered) and the location status (St-2), which the user selects via a dropdown menu. The completed dataset (User ID, St-1, St-2, **Δ*P***, **V**_**b**_) is sent to the web server in JSON format. The smartphone communicates with the web server at 2-second intervals via HTTP POST/GET using a mobile data connection.

The smartphone also receives computation results from the web server, including the current values of Saved Allowance Time (SAT) and Non-wearing Time (NWT). It displays St-1, St-2, SAT, and NWT values on-screen, along with a band-style Behavioral State Indicator, color-coded according to the user’s current behavioral state based on the combination of St-1 and St-2.

As shown in Table 1, the combination of Wearing Status (St-1) and Location Status (St-2) determines the behavioral safety level and is visually represented via the Behavioral State Colors. If St-1 = 1 (wearing), the state is shown as “safe” in green, regardless of location. If St-1 = 0 (not wearing), being in a Certified Safe Zone (CSZ, such as home) is considered safe and displayed in blue. In contrast, being outside CSZ without wearing the PAPR is deemed a high-risk violation and displayed in red. As an exception, during SAT Utilization or Exemption Mode (SAT-EX), non-wearing outside CSZ is temporarily permitted and shown in purple.

This figure presents the actual appearance of the prototype PAPR mounted on a mannequin, along with the display screen of the corresponding smartphone application. The PAPR adopts a helmet-integrated structure and enables wearing status detection using a differential pressure sensor. The smartphone application provides intuitive visual feedback to the user by displaying color-coded bands according to the Wearing Status (St-1) and Location Status (St-2), allowing the user to instantly recognize their current behavioral state (e.g., safe, violation, or SAT utilization).

### Web server

The web server, implemented as a Perl CGI program, receives the datasets transmitted from smartphones and is responsible for updating and managing SAT and NWT based on a rule-based protocol. The computed results are sent back to the smartphone.

SAT and NWT are saved per user ID, along with timestamps, as text files (sat.txt and nwt.txt), with time values recorded in milliseconds. Special commands are available for initialization and resetting. SAT is decremented (per millisecond) when St-1 = 0 (non-wearing) and St-2 = 0 (outside CSZ), or St-2 = 99 (SAT-EX mode). NWT is incremented by the time difference (in milliseconds) when St-1 = 0 and either St-2 = 0 or St-2 = 99 with SAT ≤ 0.

SAT and NWT updates are automatically processed with each dataset received from the smartphone, and the updated values are returned to the smartphone and stored on the server.

**Table 1:**
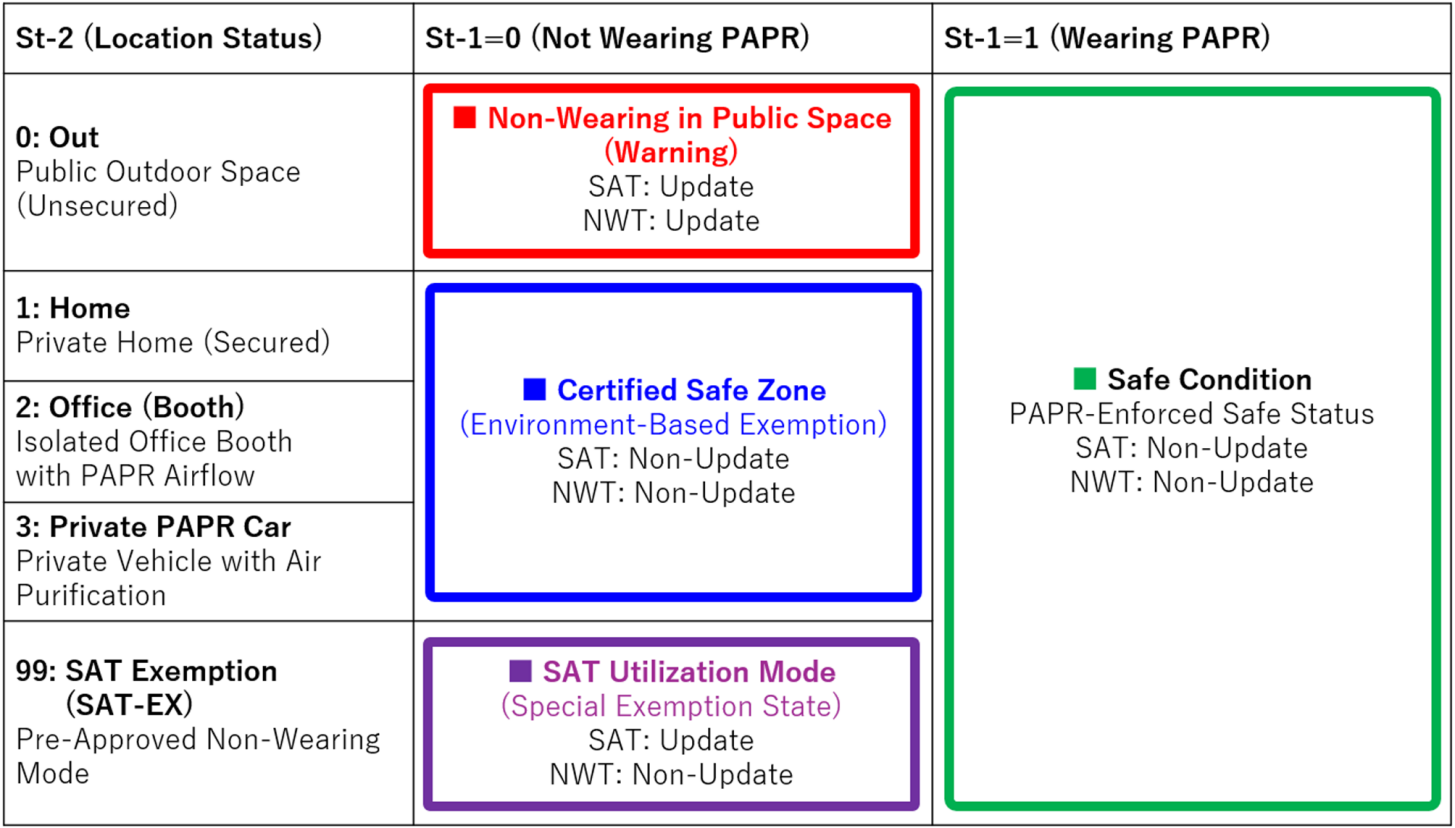
Behavioral state colors based on wearing status (St-1) and location status (St-2). Combination of system-detected Wearing Status (St-1) and user-declared Location Status (St-2) defines the safety level and determines whether SAT or NWT should be updated. Color-coded regions match system feedback shown in the smartphone application.

## Validation of the PWS-NET prototype system under realistic behavioral scenarios

Validation experiments of the prototype PAPR Wearing-Status Networked Management System (PWS-NET), assuming realistic behavioral scenarios, have been conducted. The objective was to verify whether updates of Saved Allowance Time (SAT) and Non-wearing Time (NWT) are performed correctly by the rule-based logic of the system in response to user behavior transitions.

As shown in Table 2, four representative user scenarios were defined: Basic Authorized Outing, User-Initiated Exemption, Resume Secured Wearing, and Mis-declared Homing. Validation was conducted using a mannequin equipped with the prototype PAPR. Notably, behaviors that would violate the rules—such as being unmasked in public spaces—were excluded from this validation, as the actual system is assumed to suppress such behaviors effectively through social mechanisms like checkpoints and legal penalties.

**Table 2:**
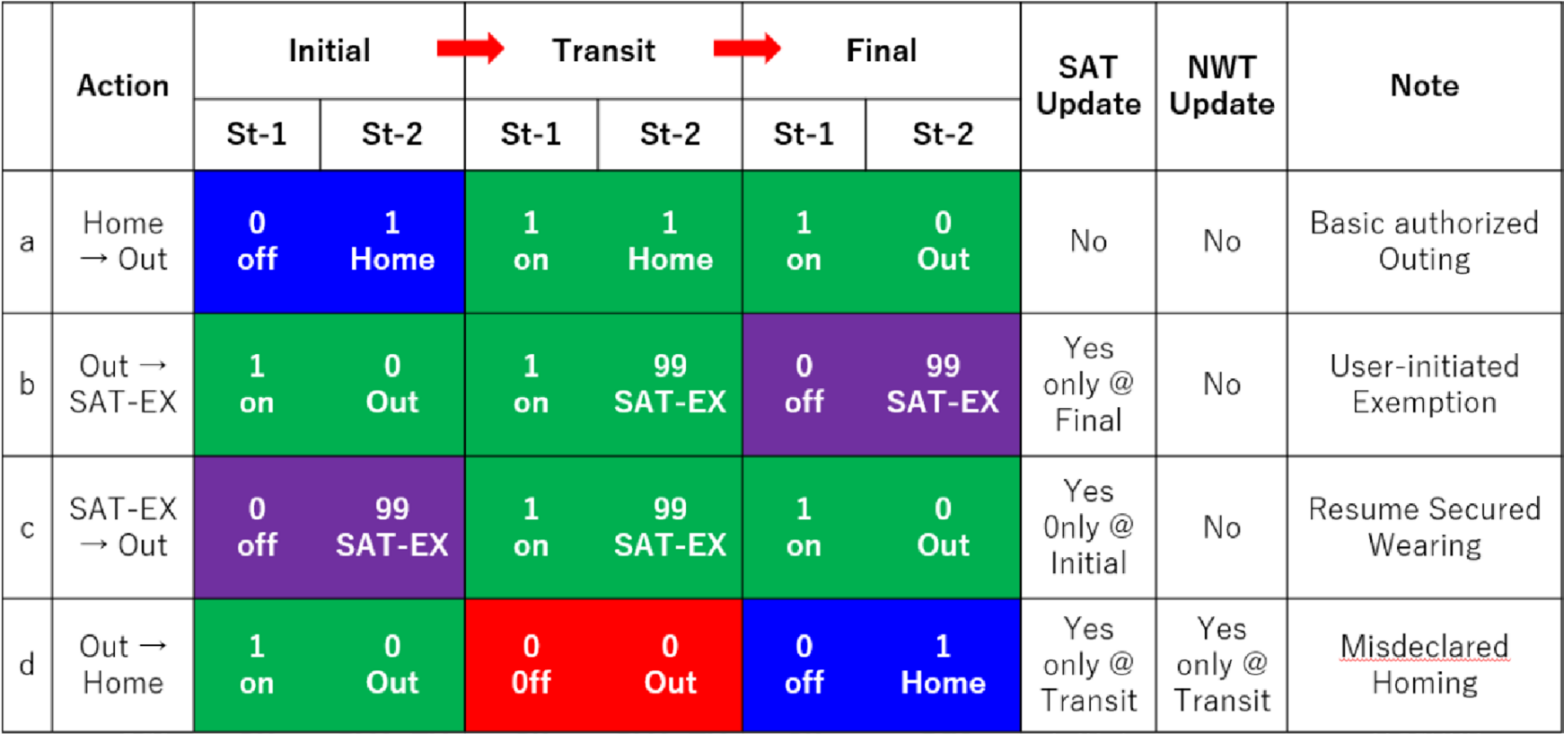
Transition scenarios and corresponding updates of SAT and NWT. Each row represents a realistic user behavior scenario, with status transitions across Initial, Transit, and Final stages. Color coding and update flags indicate whether the scenario triggers changes in SAT or NWT.

Table 2 summarizes the transitions in Wearing Status (St-1) and Location Status (St-2) for each scenario (Initial state, Transit state, Final state), and the resulting color-coded Behavioral State Indicator derived from each combination: green (safe: wearing), blue (safe: unmasked in CSZ), red (violation: unmasked outside CSZ), and purple (SAT-EX: SAT utilization mode). Based on these combinations, the system determines whether SAT or NWT should be updated. The rightmost column shows the update flags (YES = update, NO = no update). The initial condition was set as (SAT, NWT) = (3600 s, 0 s).

**Fig. 4:**
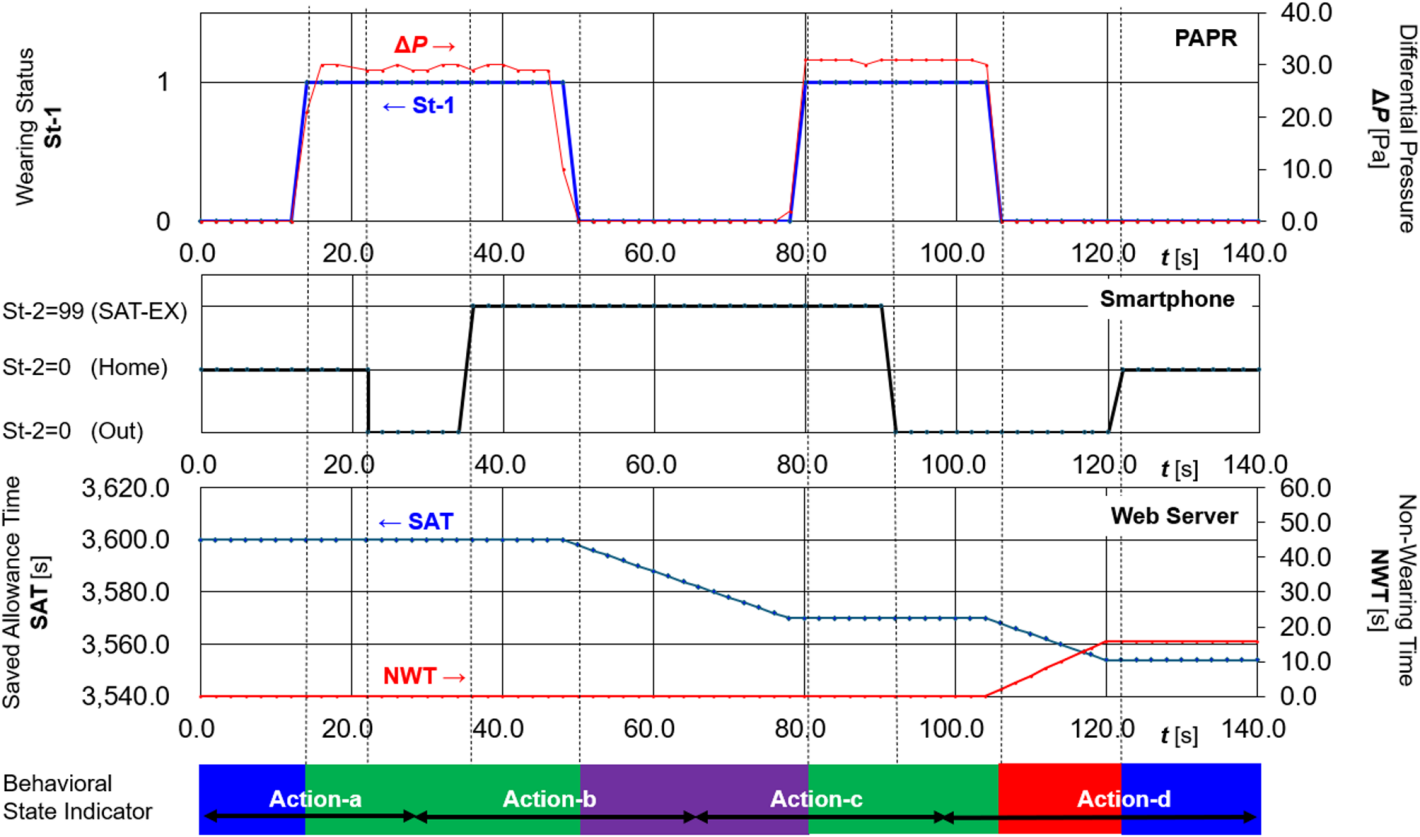
Time-series transitions in St-1, St-2, differential pressure (Δ*P*), SAT, and NWT with corresponding behavioral state color changes. Time-series plots showing transitions in Wearing Status (St-1), Location Status (St-2), differential pressure (**Δ*P***), and the corresponding updates in Saved Allowance Time (SAT) and Non-wearing Time (NWT). The system accurately reflects behavioral conditions and updates the time budgets accordingly.

Figure 4 shows the experimental results as time series data for St-1, St-2, differential pressure (**Δ*P*** ), SAT, NWT, and the color of the Behavioral State Indicator. The following observations were made:

1. St-1 (wearing status), derived from differential pressure sensor values, was accurately determined in real time.
2. St-2 (location status) was manually selected on the smartphone.
3. SAT and NWT were updated with precise timing as defined by the rule set.
4. The Behavioral State Indicator colors were displayed accurately as designed.

These results indicate that the system reliably tracks behavioral transitions in real time and stably manages time-based parameters in accordance with the rule-based logic.

**Fig. 5:**
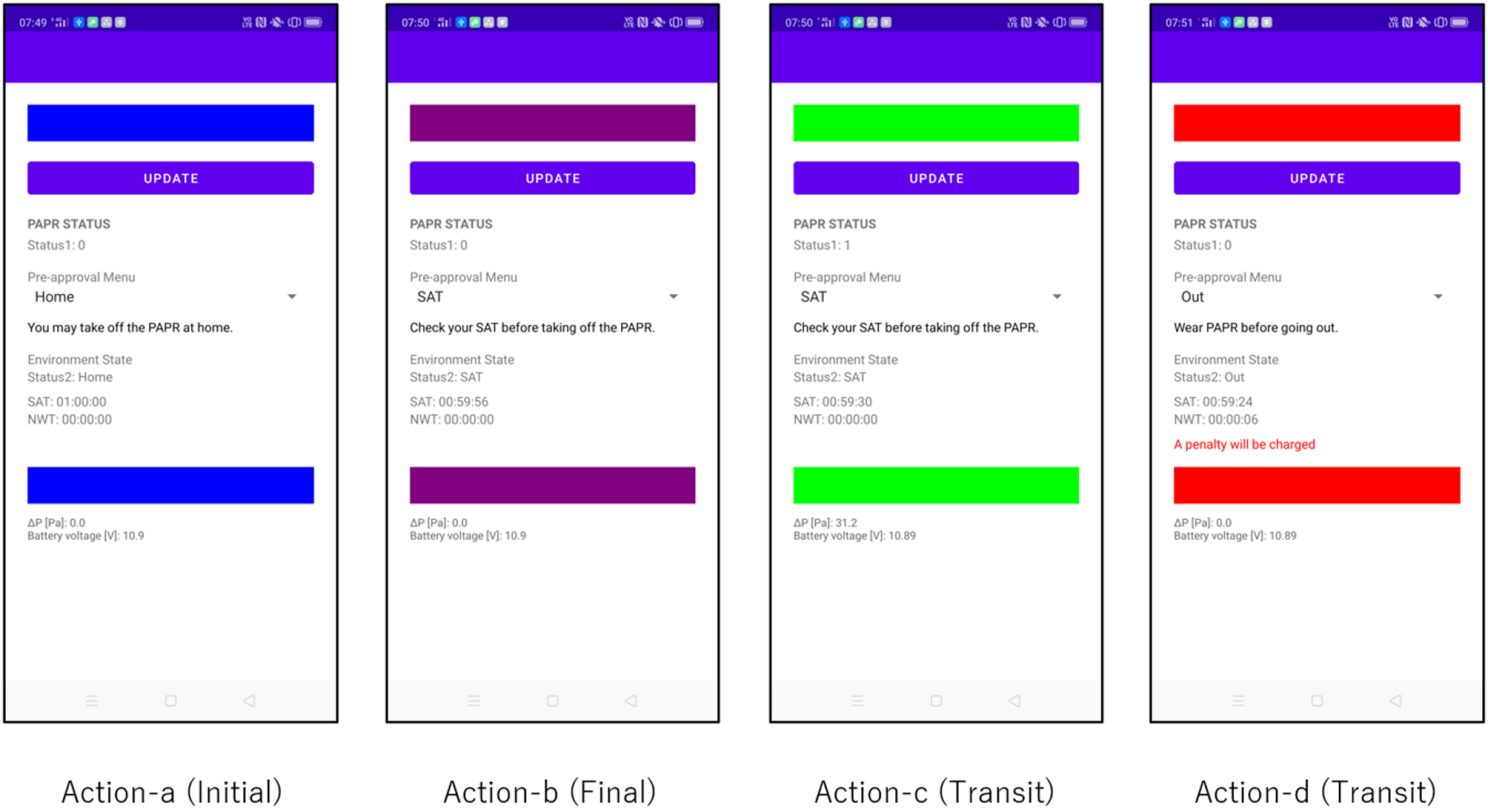
Experimental results: screenshots of the mobile application interface during representative transitions. The interface reflects real-time St-1 and St-2 values, alerts, and SAT/NWT status. Color-coded backgrounds correspond to system interpretations: safe (green), exempted (purple), and violation (red).

Figure 5 presents screenshots of the smartphone application at key transition points. The Behavioral Color Indicator clearly represents the current behavioral state—green (safe: wearing), blue (safe: unmasked in CSZ), red (violation), and purple (SAT-EX)—providing intuitive feedback to the user. Although not easily visible in the figure, the app displays St-1, St-2 (dropdown menu selection), SAT/NWT (hh: mm: ss format), differential pressure (**Δ*P***), and battery voltage (**V**_**b**_). Additionally, in Action-d (Transit), when NWT exceeds zero, a red warning message stating “A penalty will be charged” is shown.

The application also displays appropriate alerts and behavioral instructions, allowing the user to intuitively understand the system’s decisions. These GUI behaviors demonstrate sufficient reliability and visibility to support real-world use, effectively guiding user actions.

The prototype system successfully executed SAT and NWT update processes without error or malfunction under all tested realistic scenarios. The update decisions were handled exactly as intended by the server-side CGI program, based on rule-based logic using the system-detected value St-1 and the user-declared value St-2. These scenario-based validations demonstrate that PWS-NET can function as a practical SAT management tool and confirm that the foundation for future real-world implementation is steadily being established.

To illustrate the system’s rule-based behavior under realistic usage, we simulated four representative movement patterns in which the participant (the author) performed transitions across allowed and exempted environments while continuously wearing the PAPR. In all cases, the system correctly maintained or updated SAT and NWT values in accordance with the defined logic.

In this prototype study, scenarios triggering the penalty mode (St-1 = 0 and St-2 = 0) were excluded, as the real-world implementation assumes strict enforcement through checkpoints or mandatory mobile/PAPR use, effectively deterring unauthorized non-wearing behaviors.

## Discussions

### Positioning of this study in relation to prior research

This study is grounded in the original concept of “PAPR for everyone as an alternative to lockdown”. To the best of our knowledge, this idea was first proposed by the present author. While its logical validity has been partly recognized through academic publications and a granted patent, broad social recognition remains limited. In prior work, the author (i) proposed that medical Powered Air-Purifying Respirators (PAPRs) could be utilized by the general public as an alternative to lockdowns^11^, (ii) developed and disclosed a self-made prototype PAPR that achieved aerosol-blocking and airflow performance equivalent to medical devices at an approximate cost of USD 40^11^, and (iii) demonstrated through mathematical modeling that if more than 55% of the population wears PAPRs continuously—or if everyone wears them intermittently to a moderate extent—the effective reproduction number (***R***_**t**_) under conditions of ***R***_**t**_ = 2.0 can be reduced to ≤ 0.9^12^.

Face masks have been promoted as part of the “New Lifestyle” approach, but they are structurally limited by negative pressure during inhalation, which allows ambient air to leak through gaps. In contrast, PAPRs maintain a positive internal pressure, thereby effectively preventing the inflow of external aerosols. This positive-pressure mechanism provides particularly high protection in helmet/hood-type PAPRs; when performance is guaranteed by the manufacturer (e.g., through WPF or SWPF testing), the Assigned Protection Factor (APF) can reach as high as 1000^27^. This is orders of magnitude greater than the protection level of a half-mask respirator (APF = 10) or an N95 mask (also APF ≈ 10). According to OSHA, the APF is defined as the ratio of external to internal concentration^27^, and its application requirements are regulated in OSHA standards^30^. Despite such high aerosol-blocking performance, PAPRs have so far been used almost exclusively by those working in extremely hazardous environments, and proposals for their systematic use by the general public have been virtually absent.

Against this backdrop, the present study extends the earlier concept of “PAPR for everyone as an alternative to lockdown” and proposes the PAPR Wearing-Status Networked Management System (PWS-NET) as a socio-technical framework to make “PAPR for Everyone” both more effective and more efficient in practice. PAPRs are highly protective and can be manufactured at relatively low cost, supporting the feasibility of this concept. However, the insight has emerged that “universal and continuous PAPR wearing” would be excessive. At the same time, effective governmental infection control requires broader and more granular measurement. Therefore, a sensing-network management approach that simultaneously respects the individual’s right to choose when and where not to wear a PAPR and improves governmental visibility and efficiency in infection control is warranted. PWS-NET is proposed as one such framework.

In addition, when considered in relation to other infection-control measures, “PAPR for everyone as an alternative to lockdown” is envisioned, as illustrated in Fig. 1, as a complementary non-pharmaceutical intervention (NPI) that specifically targets airborne transmission routes, supplementing other measures such as New Lifestyle practices and vaccination. Furthermore, PWS-NET is inherently compatible with digital contact-tracing applications, suggesting potential synergies.

### Roadmap for implementation

This study remains at a basic and preliminary conceptual stage, and numerous challenges must be overcome before social implementation can be realized. Looking ahead, it appears appropriate to promote PAPR for Everyone together with the parallel promotion of PWS-NET, which is intended to enhance its effectiveness. An example roadmap for such implementation is outlined below.

As a first step, small-scale pilot studies should be conducted with a limited number of participants under realistic daily-life scenarios. These studies will allow the identification of hardware and software shortcomings and enable iterative improvement. Whereas the present work was limited to mannequin-based functional testing under constrained scenarios, future evaluations must take place under conditions closer to real-world use. For the PAPR unit itself, durability testing, improved maintainability, enhanced wearing comfort through materials and structural refinements, and long-term comfort considerations—including CO_2_ and O_2_ concentrations, temperature, humidity, and noise—are all required. In addition, the development of personalized fitting using head-shape measurements and 3D printing, the pursuit of low-cost mass-market designs, and the parallel exploration of high-performance or premium models for users willing to pay for added value will also be essential.

At the same time, the definitions and update rules of SAT and NWT—the core metrics of PWS-NET—must be further refined. In particular, the current approach to wearing-status detection (St-1), which relies on a simple differential-pressure threshold, should evolve toward adaptive estimation methods capable of handling diverse real-world usage patterns. In this study, St-1 estimation was described only qualitatively; in future work, quantitative performance indicators such as detection accuracy and false-positive/false-negative rates will need to be incorporated.

The next stage will be larger-scale proof-of-concept trials, such as the distribution of thousands of PAPRs within a community, coupled with statistical comparisons. Such trials will help clarify the actual public-health effectiveness of PAPR for Everyone and the added value of PWS-NET.

Building on these results, the ultimate aim will be a phased progression: from the discussion of epidemiological and public-health effectiveness, to formal institutional design, to industrial product development and mass production, and finally to societal implementation. Even without PWS-NET, widespread PAPR use could serve as an effective pandemic countermeasure. However, the parallel development of PWS-NET opens the possibility of achieving a higher-level balance between individual freedom and governmental infection control. For this reason, pursuing both tracks simultaneously is strongly recommended.

In summary, PAPR for Everyone is an ambitious concept that seeks to substitute for lockdowns and entails the construction of a very large socio-technical system. PWS-NET, which is envisaged as inseparable from this concept, will necessarily require a robust privacy-protection framework, making it a significant challenge. Nevertheless, the potential benefits justify this challenge, and its pursuit is of considerable social and technical significance.

### Effectiveness of PWS-NET

The primary purpose of PWS-NET is to achieve a high-level balance between individual freedom and governmental infection control. Here, infection control does not imply demanding absolute compliance from each individual; rather, the goal is to secure statistically reliable effects at the population level. In other words, inaccuracies in self-declared location status (St-2)—whether due to intentional falsification or inadvertent error—are incorporated in advance as expected occurrences, and the government sets the Allowance Time (AT) at each point in time with these rates taken into account. This framework allows infection control to be pursued without unnecessarily reducing AT, thereby preserving citizens’ freedom while still curbing epidemic spread. Furthermore, even if infection forecasts prove significantly inaccurate, the government retains the option of setting AT to zero, thereby mandating continuous PAPR use in public spaces. This ensures a rapid fallback mechanism to regain control, meaning that excessive precaution is not required.

To address falsification of St-2 declarations, random inspections and stricter penalties could be combined with technical enhancements such as GPS or geofencing to verify reported locations. However, such technical measures involve trade-offs with privacy, necessitating careful institutional design. Even so, the credibility of the self-reporting system can likely be maintained at a sufficient level through the combined application of spot checks and penalties. This reliability should be continually examined and reinforced through social experiments.

Because PAPRs can physically block airborne transmission routes, demonstrating through experimental and field studies that “continuous PAPR wearing in public spaces” can serve as an alternative to lockdown (i.e., restrictions on leaving home) would be of great significance for governmental infection control. Once PAPRs become widespread, governments would be able to test various infection-control hypotheses with greater flexibility and assurance.

Moreover, the SAT system itself can be further refined. In the present study, the consumption rate of SAT during exemptions (SAT-EX) was assumed to be uniform, such that one hour of non-wearing corresponds to one hour of SAT consumption. In actual practice, however, introducing weighting factors based on location and risk levels would be more realistic. For example, an outdoor walk might be assigned a coefficient of 0.5 (so that one hour of non-wearing consumes only 30 min of SAT), dining in a sparsely populated restaurant might be weighted at 1, while participation in a crowded indoor event could carry a coefficient of 2 (1 h of non-wearing consuming two hours of SAT).

As an alternative to SAT, a new indicator based on estimated virus intake could also be introduced. Specifically, by combining estimates of environmental viral concentration, individual respiratory flow rate, and the protective capacity of the PAPR, one can estimate the amount of virus inhaled per unit time with or without PAPR use. By setting allowable thresholds for the cumulative intake, infection-control governance could be made more risk-based and flexible.

In practical operation, ensuring data quality will also be a critical issue for the accurate management of SAT and NWT. Mechanisms such as time-stamp synchronization, integrity checksums, loss/error logging, and periodic sensor calibration are required to secure the precision of time management and to prevent drift or misclassification during long-term operation.

### Privacy protection and governance

If PWS-NET were to be implemented, it could involve the handling of massive amounts of big data, including not only detailed location and behavioral information of all citizens but also health-related data such as coughing patterns. The scope of personal data to be managed is therefore a critical issue. At a minimum, the system must process each individual’s whereabouts and PAPR wearing status, and without guarantees of comprehensive privacy protection, such a system would be unlikely to gain social acceptance in democratic states. Conversely, if rigorous privacy protection can be achieved, PWS-NET could be positively accepted and socially implemented as a system capable of reconciling, at a high level, the individual’s freedom to choose when and where to be without a PAPR and the government’s need for infection control. At present, it is no exaggeration to say that the greatest challenge for realizing PWS-NET in democratic societies lies in securing such privacy protection.

Of course, PAPR for Everyone could be operated even without PWS-NET. However, in that case, the system would inevitably be applied in a rigid manner that excessively restricts personal freedom, while governments would be unable to track individuals’ behaviors and wearing conditions. This would limit both the precision of infection modeling and the efficiency of infection control. Thus, it is of substantial importance to focus efforts on achieving strong privacy protection within PWS-NET so that it can obtain social acceptance.

There are two principal risks of misuse regarding data handled by PWS-NET. The first is unauthorized external access (e.g., hacking), while the second is intentional or arbitrary misuse by authorized insiders such as administrators, operators, or data owners. For the first type of threat, existing security technologies—including access control, authentication, encryption, and intrusion-detection systems—are considered effective. In addition, privacy-preserving data mining (PPDM) has been proposed as an approach that removes or transforms personally identifiable information from datasets^31^. PPDM includes techniques such as perturbation, anonymization, encryption, secure multiparty computation, and stream-data processing, but these methods face practical limitations, such as trade-offs between privacy protection and data accuracy, as well as high computational overhead.

The second type of threat—misuse by authorized insiders—is more deeply rooted and structural. System administrators and operators, by virtue of their legitimate access rights, could arbitrarily manipulate or repurpose data. This cannot be fully prevented through access control or log monitoring alone. In fact, issues such as log tampering, ambiguous guidelines, and the absence of robust audit functions have been highlighted as institutional vulnerabilities. In response, recent discussions have emphasized the importance of making data-use processes externally verifiable^32^ and establishing institutional and societal frameworks for oversight through mechanisms such as independent audits and citizen participation^33,34^.

To address such insider threats, this study considers the author’s proposed framework of a Verifiable Record of AI Output (VRAIO)^35^ as a promising option. In VRAIO, AI systems handling privacy-sensitive data are placed behind a firewall managed by a third-party body called a Recorder. Any data output from the AI must be submitted as a request to the Recorder, which receives only the purpose and summary of the proposed output rather than the data itself. The Recorder, without accessing the underlying data, evaluates the request and determines whether to approve or reject the output. Furthermore, all output requests and decisions—including (i) the submitted purposes and summaries, and (ii) the corresponding approval or rejection results—are stored in an immutable, verifiable format. This ensures that third parties can retrospectively audit whether the data actually released by the AI is consistent with the originally approved purpose and summary.

In addition, the framework combines technical and institutional measures to deter fraudulent requests. For instance, individuals or organizations (audit bodies, bounty hunters, or ordinary citizens) who detect misconduct would receive substantial rewards, while violators such as AI operators or managers would face severe penalties. By coupling (a) a technical infrastructure that records all output purposes and summaries in an immutable and verifiable form with (b) a social incentive structure of rewards for whistleblowers and penalties for violators, the system can be institutionally designed to ensure that AI outputs are restricted to those that have been socially authorized in advance.

It must be acknowledged, however, that VRAIO represents a very large-scale socio-technical system, and its construction would entail significant social costs. Nevertheless, in an increasingly information-intensive and AI-driven society, if strong privacy protection can be institutionally secured, it would enable the preservation of democratic values while simultaneously allowing society to benefit from advanced technologies. For this reason, pursuing stringent privacy protection within PWS-NET is itself of high value.

In conclusion, the social acceptance of PWS-NET is contingent upon robust privacy protection. Although its realization will be difficult, overcoming this challenge would allow citizens to benefit from PWS-NET. Moreover, the same principles could extend to future AI-driven societies, offering a pathway to preserve democratic values while enabling society as a whole to enjoy the benefits of technological advancement.

### Future extensions and technical improvements

The differential pressure sensor introduced in this study offers further opportunities for functional extension. For example, by analyzing differential-pressure data in real time, coughing and abnormal breathing could be detected, enabling early identification of possible infection symptoms. In addition, improvements to the PAPR itself could incorporate dynamic pressure control that adjusts airflow according to inhalation and exhalation phases (increasing pressure during inhalation and reducing pressure during exhalation), thereby providing a form of respiratory assistance to the wearer^36,37^.

The PAPR also holds considerable potential for expansion as a stand-alone multifunctional device. Since it is already equipped with a head-mounted support structure and a power supply (battery), it can readily accommodate the addition of controllers, various sensors, and input/output devices. This creates opportunities for its development into a wearable platform. Specific possibilities include integration with biosensors and environmental sensors to serve as a combined health and environmental monitor, or convergence with smartphone functions, potentially even incorporating AR/VR displays. Much like smartphones today have become indispensable to modern life, highly information-integrated PAPRs could in the future become essential personal devices. Moreover, if PAPRs that are both comfortable and high-performance were commercialized, people might choose to use them daily for the benefit of “purified breathing air,” regardless of governmental mandates. In the same way that modern populations routinely consume purified drinking water—a practice that has contributed to the eradication of many waterborne diseases—breathing purified air may eventually help suppress or even eliminate airborne infectious diseases.

On the other hand, the extension of PWS-NET opens new possibilities from the perspective of big-data collection. If detailed GPS data or health-related information (such as coughing detection) were available, governments could monitor infection status more accurately and construct higher-precision epidemiological models. At the same time, however, the risks of personal privacy violations would increase, necessitating strong protective measures. Integration of PWS-NET with digital contact-tracing applications is also conceivable. For instance, Singapore’s TraceTogether system demonstrated early success^38^, but was later criticized due to privacy concerns stemming from police access to the data^39^. By contrast, the Apple/Google Exposure Notification API employed a decentralized architecture with stringent privacy protections^40^; however, authorities could not assess the number of notifications issued or the degree of behavioral change induced, leading to doubts about its effectiveness^41^. This illustrates the unavoidable trade-off between privacy and effectiveness. The application of the Verifiable Record of AI Output (VRAIO) framework^35^ could provide a promising solution to this dilemma.

If robust privacy protection could be realized in PWS-NET, it would become possible to collect highly detailed personal data at scale, thereby enabling further refinement of infection models and more precise infection control based on those models. Furthermore, such an approach could be extended beyond PWS-NET to other IoT/ICT and AI systems. By handling vast amounts of personal information as big data while ensuring strong privacy protection, society could move toward a highly efficient and safe future infrastructure. In this sense, it is no exaggeration to state that the extent of privacy protection will determine the scope of utilization of PWS-NET.

## Conclusions

In this study, under the framework of the concept “PAPR for everyone as an alternative to lockdown”—which, to the best of our knowledge, was first proposed by the author—we aimed to reconcile citizens’ freedom to choose when and where not to wear a PAPR with the efficiency of governmental infection control. To this end, we proposed a new socio-technical framework, the PAPR Wearing-Status Networked Management System (PWS-NET), and through prototyping and operational testing, we preliminarily confirmed both its technical feasibility and its institutional viability.

PWS-NET integrates real-time detection of PAPR wearing status, user-declared location, and rule-based decision-making by a web server to dynamically manage Saved Allowance Time (SAT), thereby functioning as an IoT-based system designed to harmonize individual freedom with infection-control measures. In evaluation experiments simulating representative behavioral scenarios using a mannequin, SAT updates were executed accurately according to predefined rules, and visual feedback to users functioned as intended.

These results indicate that PWS-NET represents a practical framework with the potential for future social implementation, while also serving as a starting point for large-scale field trials and institutional design. Importantly, the system exhibits significant potential for extensibility, including the introduction of weighted SAT control, the use of alternative indicators such as Estimated Virus Dose (EVD), integration with the Verifiable Record of AI Output framework to safeguard privacy, and the addition of functions such as exposure notification and respiratory diagnostics.

Looking forward, in order to enhance the social acceptability of the “PAPR for everyone” concept and of PWS-NET, comprehensive empirical research is needed that incorporates not only technical reliability but also ethical, legal, and institutional perspectives, alongside the development of phased strategies for real-world deployment. By embedding the Verifiable Record of AI Output framework, PWS-NET has the potential to serve as a core technology in a new model of pandemic countermeasures—one capable of achieving a high-level reconciliation between individual freedom and governmental infection control—and to play an important role in future public-health governance.

## Data Availability

The datasets used and/or analyzed during the current study are available from the corresponding author (Yusaku Fujii, fujii@gunma-u.ac.jp) on reasonable request.
